# Combination of laser and human adipose-derived stem cells in repair of rabbit anal sphincter injury: a new therapeutic approach

**DOI:** 10.1186/s13287-019-1477-5

**Published:** 2019-12-02

**Authors:** Arash Sarveazad, Asrin Babahajian, Abazar Yari, Chris K. Rayner, Marjan Mokhtare, Arash Babaei-Ghazani, Shahram Agah, Bahar Mahjoubi, Jebreil Shamseddin, Mahmoud Yousefifard

**Affiliations:** 1 Colorectal Research Center, Iran University of Medical Sciences, Tehran, Iran; 2 Liver and Digestive Research Center, Research Institute for Health Development, Kurdistan University of Medical Sciences, Sanandaj, Iran; 3 Department of Anatomy, Faculty of Medicine, Alborz University of Medical Sciences, Karaj, Iran; 4 Discipline of Medicine, University of Adelaide, Adelaide, SA Australia; 5 Centre of Research Excellence in Translating Nutritional Science to Good Health, University of Adelaide, Adelaide, SA Australia; 6 Neuromusculoskeletal Research Center, Department of Physical Medicine and Rehabilitation, Iran University of Medical Sciences, Tehran, Iran; 7 Infectious and Tropical Diseases Research Center, Hormozgan Health Institute, Hormozgan University of Medical Sciences, Bandar Abbas, Iran; 8 Physiology Research Center, Iran University of Medical Sciences, Tehran, Iran

**Keywords:** Fecal incontinence, Lasers, Mesenchymal stem cells

## Abstract

**Background:**

Anal sphincter injury leads to fecal incontinence. Based on the regenerative capability of laser and human adipose-derived stem cells (hADSCs), this study was designed to assess the effects of co-application of these therapies on anal sphincter recovery after injury.

**Design:**

Male rabbits were assigned to equal groups (*n* = 7) including control, sphincterotomy, sphincterotomy treated with laser (660 nm, 90 s, immediately after sphincterotomy, daily, 14 days), hADSCs (2 × 10^6^ hADSCs injected into injured area of the sphincter immediately after sphincterotomy), and laser + hADSCs. Ninety days after sphincterotomy, manometry and electromyography were performed, sphincter collagen content was evaluated, and Ki67, myosin heavy chain (MHC), skeletal muscle alpha-actin (ACTA1), vascular endothelial growth factor A (VEGFA), and vimentin mRNA gene expression were assessed.

**Results:**

The laser + hADSCs group had a higher resting pressure compared with the sphincterotomy (*p* < 0.0001), laser (*p* < 0.0001), and hADSCs (*p* = 0.04) groups. Maximum squeeze pressure was improved in all treated animals compared with the sphincterotomized animals (*p* < 0.0001), without a significant difference between treatments (*p* > 0.05). In the laser + hADSCs group, motor unit numbers were higher than those in the laser group (*p* < 0.0001) but did not differ from the hADSCs group (*p* = 0.075). Sphincterotomy increased collagen content, but the muscle content (*p* = 0.36) and collagen content (*p* = 0.37) were not significantly different between the laser + hADSCs and control groups. Laser + hADSCs increased ACTA1 (*p* = 0.001) and MHC (*p* < 0.0001) gene expression compared with laser or hADSCs alone and was associated with increased VEGFA (*p* = 0.009) and Ki67 mRNA expression (*p* = 0.01) and decreased vimentin mRNA expression (*p* < 0.0001) compared with laser.

**Conclusion:**

The combination of laser and hADSCs appears more effective than either treatment alone for promoting myogenesis, angiogenesis, and functional recovery after anal sphincterotomy.

## Introduction

The anal sphincter provides both resting contractile tone and voluntary contraction for its role in closing the anal canal and maintaining fecal continence [1]. Anal sphincter injury caused by trauma (e.g., during vaginal delivery) or surgical sphincterotomy can lead to fecal incontinence (FI) [2], a condition that affects women (8.9%) more than men (7.7%), and is associated with social isolation, low self-esteem and depression, and impaired quality of life [3, 4]. Surgical repair of the anal sphincter [5] has satisfactory short-term outcomes, but recurrence is common over the longer term [6, 7]. Other treatments such as artificial sphincters or mesh may carry complications including discomfort, infection, and implant failure [8]. Bulking agents are prone to displacement, emboli formation, and granulation [9, 10]. Therefore, reconstitution of muscle tissue, utilizing stem cells that are capable of differentiating into various cell types, would appear an ideal strategy to improve long-term outcomes in FI. Human adipose-derived stem cells (hADSCs) are an easily accessible and abundant source of the stem cells (10^6^ cells/g of fat tissue) [11], with a high proliferative rate [12, 13]. The paracrine effect of hADSCs leads to anti-apoptotic, anti-inflammatory, anti-fibrotic and immunomodulatory, and angiogenesis properties [14] that cause host tissue muscle regeneration. On the other hands, their capability to differentiate into muscle fibers has been demonstrated in vitro [12, 15].

Silent satellite cells in the basement membrane of muscle fibers play an important role in muscle regeneration. A low-level laser (LLL) can activate these cells preparing the division phase and finally contributing to the muscular repair process [16, 17]. Furthermore, LLL has anti-apoptotic properties [18] and can activate fibroblasts to FGF and IGF-1 secretion [19, 20], contributing to the repair and regeneration of muscle tissue. Therefore, co-application of hADSCs and LLL may be an effective strategy for anal sphincter repair. This study was designed to assess the effects of each therapy individually, and when applied together, on anal sphincter recovery and function after experimental injury.

## Materials and methods

### Animals

Thirty-five male albino New Zealand rabbits weighing 2.5–3.0 kg were purchased from Pasteur Institute of Iran. Animals were kept in standard conditions of ambient temperature (21 ± 3 °C) and 12-h dark-light cycle, with free access to fresh water and food based on the ethical rules for care and handling of laboratory animals of Iran University Ethical Committee, code 94-04-182-27064. Animals were randomly assigned to five equal groups (*n* = 7):

1. Control group: animals received no intervention
2. Sphincterotomy group: animals underwent sphincterotomy without any other intervention
3. Laser group: animals underwent sphincterotomy and low-level laser irradiation
4. hADSCs group: animals underwent sphincterotomy and 2 × 10^6^ hADSC injection into the injured anal sphincter
5. Laser + hADSCs group: animals underwent sphincterotomy and hADSC injection into the injured anal sphincter followed by low-level laser irradiation

### Isolation and immunophenotyping of hADSCs

Human subcutaneous abdominal adipose tissue was taken from female candidates aged 25 to 35 years old and transferred to a sterile dish containing FBS, DMEM/Ham’s F-12 10%, and streptomycin/penicillin (P/S) 5%. Isolation of hADSCs followed a protocol described for our previous studies [21–24]. In brief, fat tissue was rinsed twice in P/S 1% prepared with warm PBS to remove vessels and connective tissue. The samples were then transferred to 50-ml tubes containing collagenase 0.1% and BSA 1% prepared in warm PBS for 60 min to achieve tissue digestion. After a 5-min centrifugation in 12,000 rpm to remove RBCs, the resulting pellet was suspended with RBC lysis buffer for 10 min and re-centrifuged. Finally, after a PBS wash, the cells were cultured to DMEM/Ham’s F-12 in FBS 10% and P/S 1% medium. The cell flasks were kept in an incubator at 37 °C with 5% CO_2_ and 98% humidity, and in the third passage, hADSCs were characterized with flow cytometry (CD29^+^, CD73^+^, CD105^+^, CD34^−^, and CD45^−^) (Additional file 1: Figure S1).

### Sphincterotomy model

Our model was selected to achieve the equivalent of a grade 4 anal sphincter tear [tear of the external anal sphincter (EAS), internal anal sphincter (IAS), and anal mucosa] according to Sultan’s classification [25]. Under general anesthesia with ketamine (80 mg/kg) and xylazine (10 mg/kg), animals were put in the lithotomy position. The perineal skin and anus were thoroughly washed with povidone iodine and normal saline solutions, and a 1-cm incision was made with a surgical blade in the left side of the anal sphincter.

### Labeling of hADSCs

Before transplantation in the third passage, hADSCs were suspended in 1 ml PBS, and 5 μl of Dil solution (containing 50 μg Dil powder in 50 μl DMSO) was added and the cells were incubated for 5 min at 37 °C with 5% CO_2_ and 98% humidity, and then for 20 min at 8–10 °C.

### hADSC administration and LLL irradiation

In the hADSCs group, 2 × 10^6^ hADSCs/40 μl PBS was injected immediately after sphincterotomy into the injured sphincter within 2 min using a Hamilton syringe equipped with a 25-gauge needle. In the LLL group, a CW laser diode with 660-nm wavelength and 100-W power (Heltschl, model ME-TL10000-SK) was mounted on a metal rod to maintain a distance of 2 cm between the radiation source and the target site. Irradiation commenced (90 s) immediately after sphincterotomy and hADSC transplantation and was repeated daily for 14 days.

### Manometry

Ninety days after sphincterotomy, anal sphincter manometry was performed by means of a standard 4.7-mm anorectal catheter and a pressure transducer (Mui Scientific, Canada) by an operator blinded to the treatment allocation. The probe was inserted into the animal rectum without any anesthesia induction, and the balloon baseline pressure was established; then, the probe was withdrawn at a constant rate of 0.05 cm/s, and the sphincter pressure profile (resting and maximum squeeze pressures) was recorded. When the probe was inserted into the animal rectum, we stimulate the external anal sphincter with tingling (natural stimulus) and squeezing pressure was recorded. The procedure was repeated at least three times for each animal.

### Electromyography

Electromyography (EMG) was also performed 90 days after sphincterotomy using a Synergy on Nicolet EDX system (Natus Medical Corporation, USA), with adhesive electrodes applied to a 0.02-mm^2^ recording area in the hairless part of the rabbit’s earlobe, with a disposable needle (30 gauge, 0.3-mm diameter, 25 mm long; Ambu Copenhagen, Denmark). The animal was fixed in the lithotomy position without anesthesia or muscle relaxants. The EMG needle was inserted perpendicularly into the anal skin to a depth of 5 mm, adjacent to the mucosal border. The EMG sweep and sensitivity were set at 10 ms/cm and 100–200 mV, respectively. Afterwards, the number of motor unit action potentials (MUAPs) was assessed within a 20-s time window.

### Histological assessment

Ninety days after sphincterotomy, three randomly selected animals from each group were sacrificed with intracardial perfusion with 4% paraformaldehyde under deep anesthesia (80 mg/kg ketamine and 10 mg/kg xylazine). The anal sphincter was excised intact, fixed overnight with 4% paraformaldehyde, set in paraffin, and cut into 10-μm transverse serial sections. The remaining four animals in each group were deeply anesthetized in the lithotomy position, and the anal sphincter was excised and transferred fresh into cold PBS then to a − 80 °C freezer for ensuing real-time PCR technique.

### Mallory’s trichrome staining and quantitative assay of muscle and collagen content

Three sections from each animal (*n* = 3 in each group) were selected and stained with Mallory’s trichrome method. After imaging under a light microscope (× 40), the area occupied by Mallory’s trichrome stain (blue color) was measured using ImageJ software (Fiji 1.46), and the percentage of the total area positive for collagen and muscle was calculated by dividing Mallory’s trichrome area by the total area of the section [22].

### Immunohistochemistry

Ki67 primary antibody (Thermo Fisher Scientific, MA5-14520), myosin heavy chain (MHC) primary antibody (Sigma, M4276), skeletal muscle alpha-actin (ACTA1) primary antibody (Sigma, A5228), vascular endothelial growth factor A (VEGFA) primary antibody (Abcam, ab1316), and vimentin primary antibody (Sigma, V6630) were used. Alexa Fluor 594 goat anti-mouse IgG (Biolegend, 405326) was used to detect the primary antibody. Briefly, after antigen retrieval (citrate buffer pH = 6, temperatures below the boiling point, 10 min) and washing (PBS three times), a 15-min incubation with H_2_O_2_ 10% diluted in methanol was performed. After washing (PBS three times), blocking in goat serum 10% (Sigma, USA) for 30 min at 37 °C was performed. After incubation of the samples with the primary antibody (24 h at 4 °C, humidified environment) and washing (PBS three times), the samples were incubated (2 h in the dark, 37 °C, humidified environment) with the secondary antibody. Finally, DAPI staining was performed to stain the cell nuclei. The slides were photographed using a fluorescence microscope equipped with a camera (× 10).

### Gene expression analysis by real-time PCR

The total RNA was extracted using a RNA extraction kit (EURx Company, Poland) followed by reverse transcription of RNA using EURx cDNA synthesis kits and a random hexamer primer. Using the Cybergreen kit (Qiagen Company, USA), the primers of ACTA1, glyceraldehyde-3-phosphate dehydrogenase (GAPDH), MHC, VEGFA, vimentin, and Ki67 were used to analyze the gene expression using ABI StepOnePlus™ Real-Time PCR (Applied Biosystems Company, USA). Gene expression data were normalized using the GAPDH gene as an internal control. The sequence of primers is shown in a supplementary file (Additional file 1: Table S1).

### Statistical analysis

Data were analyzed in SPSS 21.0 and presented as means and standard errors. Two-way repeated measures ANOVA was used to compare the mean of the manometric findings. One-way ANOVA was used to assess the histological assays. Bonferroni post hoc was applied in all analyses and *p* < 0.05 was considered significant.

## Results

### Resting pressure and maximum squeeze pressures

**Fig. 1:**
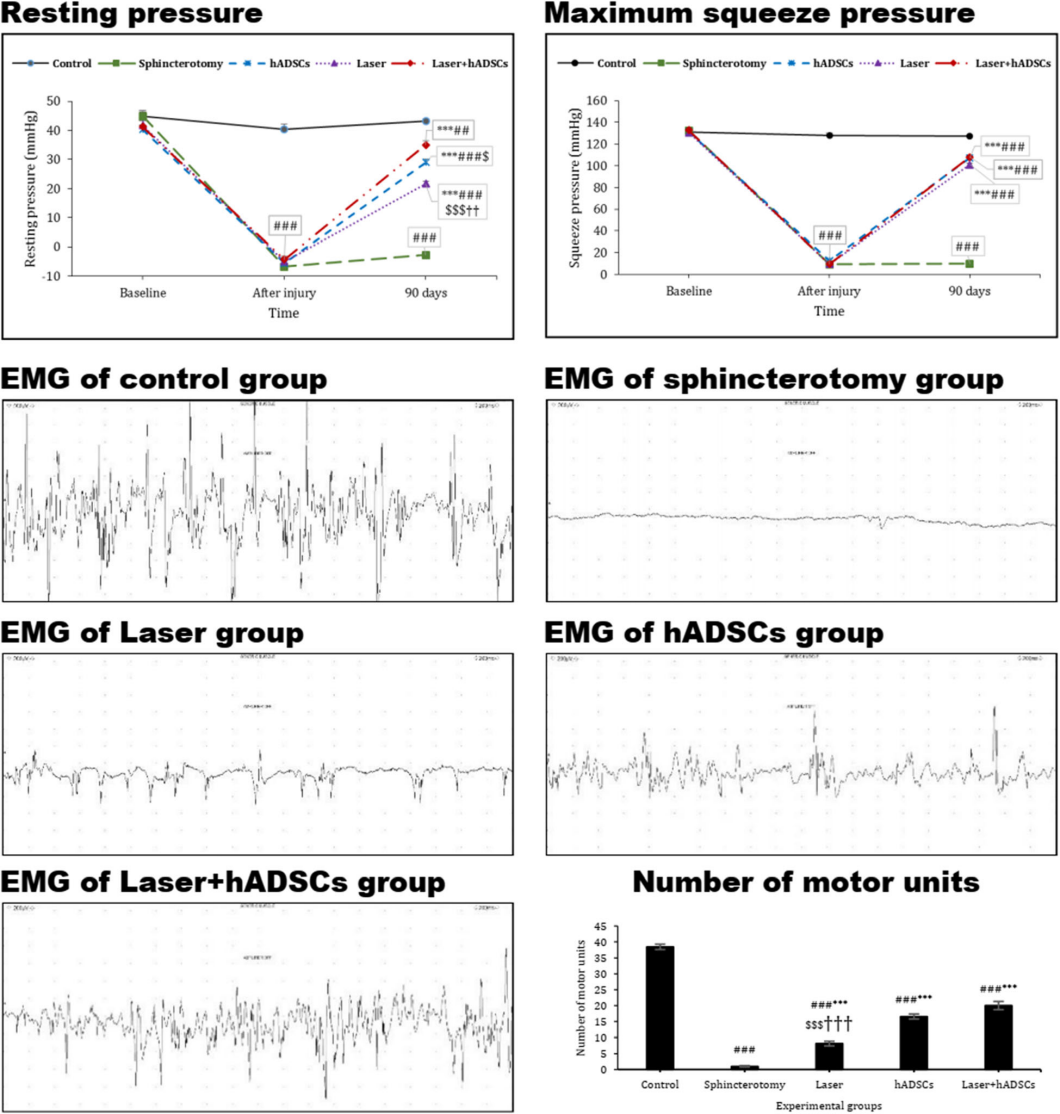
Effect of laser, human adipose-derived stem cells (hADSCs), and combination of laser and hADSCs (laser + hADSCs) on resting and squeeze anal sphincter pressures and number of motor units in the anal sphincter based on electromyography. Data are presented as means ± SEM (*n* = 7 animal per group). ***Significant level at *p* < 0.0001 with the sphincterotomy group. ^###^Significant level at *p* < 0.0001 with the control group (intact animals). ^##^Significant level at *p* < 0.01 with the control group. ^$$$^Significant level at *p* < 0.0001 with the laser + hADSCs group. ^$^Significant level at *p* < 0.05 with the laser + hADSCs group. ^††^Significant level at *p* < 0.01 with the hADSCs group

As shown in Fig. 1, sphincterotomy led to a significant decrease in resting pressure (df = 8, 60; *F* = 81.6; *p* < 0.0001). Three months after sphincterotomy, the resting pressure significantly increased in the hADSCs (29.0 ± 1.2), laser (21.7 ± 0.8), and laser + hADSCs (35.0 ± 1.4) groups (*p* < 0.0001) but did not reach the level of the non-sphincterotomy control (43.3 ± 0.8) group (*p* < 0.01). The resting pressure in the hADSCs group was higher than that in the laser-treated group (*p* = 0.05). In addition, it was higher in the laser + hADSCs group than in either the laser (*p* < 0.0001) or hADSCs (*p* = 0.04) groups.

The maximum squeeze pressure in sphincterotomized animals (10.0 ± 0.6) was dramatically decreased (df = 8, 60; *F* = 400.3; *p* < 0.0001). After 3 months, maximum squeeze pressure increased in the laser (101.1 ± 1.4), hADSCs (107.4 ± 1.2), and laser + hADSCs (108.2 ± 2.2) groups (*p* < 0.0001) but did not reach to controls (127.4 ± 1.2) (*p* < 0.0001). No significant difference in maximum squeeze pressure was seen between the laser (*p* = 0.06), hADSCs (*p* > 0.99), and laser + hADSCs groups (Fig. 1).

### Motor unit numbers

EMG testing showed motor unit number reduction in the sphincterotomy group (df = 4, *F* = 280.4; *p* < 0.0001). The laser (8.1 ± 0.7; *p* < 0.0001), hADSCs (16.6 ± 0.7; *p* < 0.0001), and laser + hADSCs groups (20.0 ± 2.2; *p* < 0.0001) displayed substantially more motor units than the sphincterotomy group (1.0 ± 0.2; *p* < 0.0001), with a higher number in the hADSCs (*p* < 0.0001) and laser + hADSCs (*p* < 0.0001) groups than in the laser group, without any difference between hADSCs and laser + hADSCs (*p* = 0.075) (Fig. 1).

### Collagen content in the injured sphincter

**Fig. 2:**
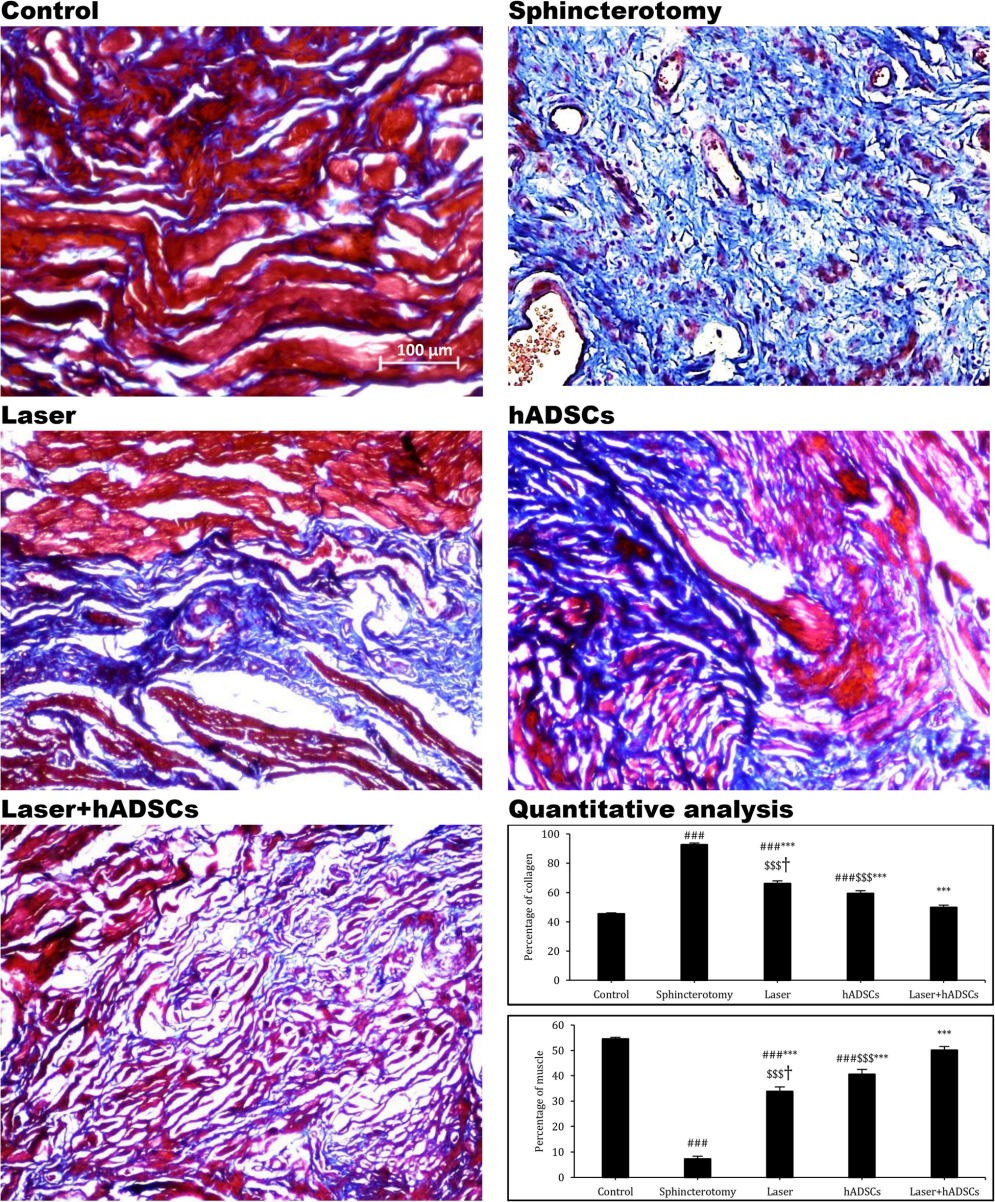
Amount of collagen and muscle tissues in injury site of the external anal sphincter after laser, human adipose-derived stem cells (hADSCs), and combination of laser and hADSCs (laser + hADSCs) treatments. Data are presented as means ± SEM (*n* = 3 animal per group). ***Significant level at *p* < 0.0001 with the sphincterotomy group. ^###^Significant level at *p* < 0.0001 with the control group (intact animals). ^$$$^Significant level at *p* < 0.0001 with the laser + hADSCs group. ^†^Significant level at *p* < 0.05 with the hADSCs group. Mallory’s trichrome staining, external anal sphincter, rabbit (× 20)

The collagen content, which was reduced in all treatment groups (92.7 ± 1.1; df = 4, *F* = 162.8; *p* < 0.0001), increased 3 months after sphincterotomy. Among the treatments, the hADSCs group (59.4 ± 1.9) had less collagen than the laser group (66.2 ± 1.8) (*p* = 0.02). Additionally, the laser + hADSCs group (49.9 ± 1.5) showed the least collagen content, the same as the control group (45.5 ± 0.6) (*p* = 0.37) (Fig. 2).

### Muscle tissue content in the injured sphincter

The muscle content of the anal sphincter decreased 90 days after sphincterotomy in all groups (7.3 ± 1.0; df = 4, *F* = 192.8; *p* < 0.0001). However, it was increased in all treatment groups. Among the treatment groups, the hADSCs group (40.6 ± 1.9) had more muscle content than the laser group (33.7 ± 1.7) (*p* = 0.021). It is worth mentioning that the muscle content of the laser + hADSCs group (51.1 ± 1.4) was not different from that of the control group (54.5 ± 0.7) (*p* = 0.36) (Fig. 2).

### ACTA1 gene expression

**Fig. 3:**
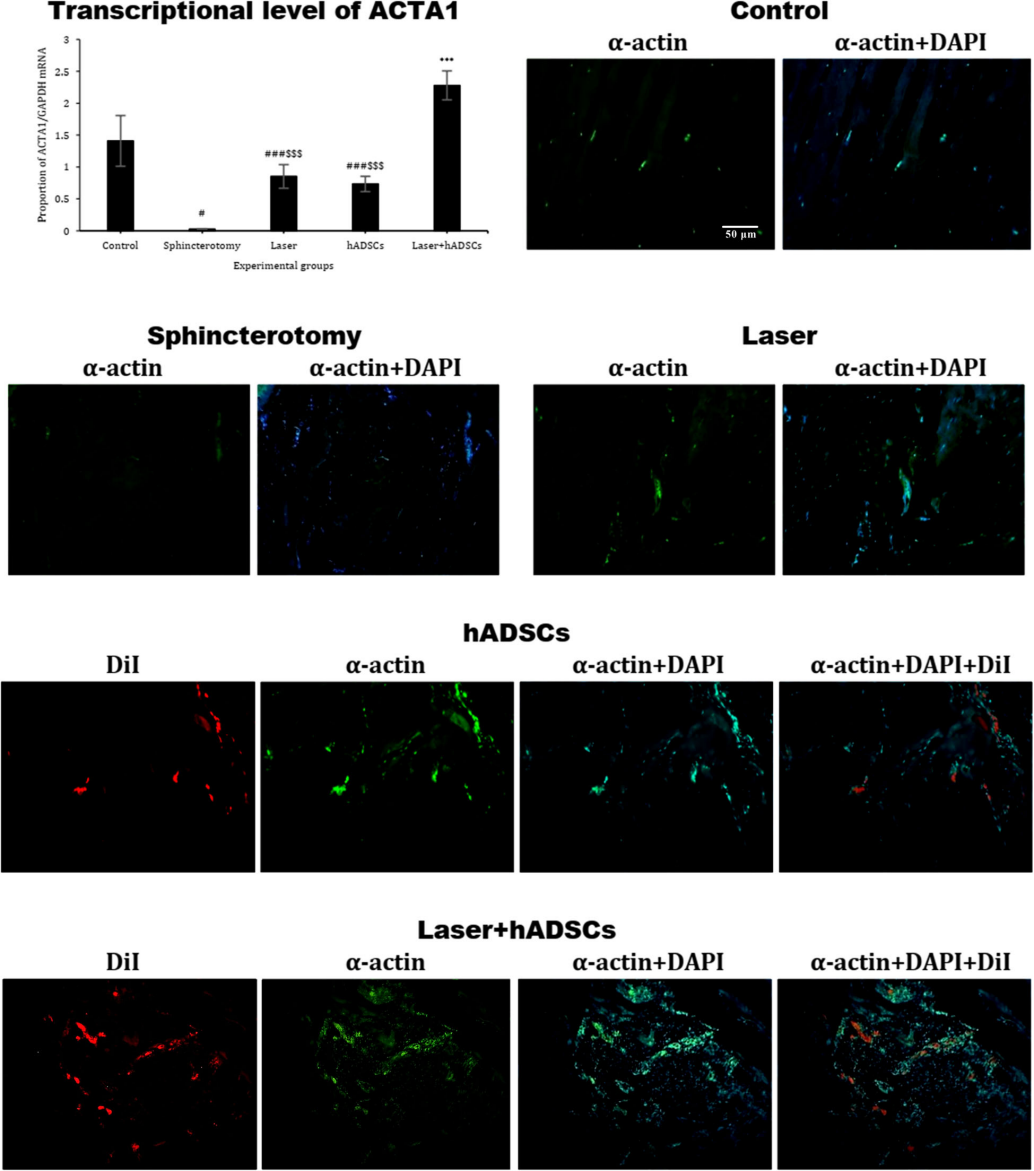
Gene and protein expression of actin in injury site of the external anal sphincter after laser, human adipose-derived stem cells (hADSCs), and combination of laser and hADSCs (laser + hADSCs) treatments. hADSCs labeled by DiI (red). α-actin expression is observed in the hADSCs and laser groups (green). Data are presented as means ± SEM (*n* = 4 animal per group). ***Significant level at *p* < 0.0001 with the sphincterotomy group. ^###^Significant level at *p* < 0.0001 with the control group (intact animals). ^#^Significant level at *p* < 0.05 with the control group (intact animals) ^$$$^Significant level at *p* < 0.0001 with the laser + hADSCs group. Real-time PCR, immunohistochemistry, and western blotting, anal sphincter, rabbit (× 10)

Real-time PCR showed that sphincterotomy (0.03 ± 0.005) decreased ACTA1 mRNA gene expression compared with the control group (1.4 ± 0.4) (df = 4, *F* = 13.4; *p* = 0.001). The laser + hADSCs group (2.3 ± 0.2) had increased ACTA1 expression compared with the sphincterotomy group (*p* = 0.001), while there was no difference between the laser (0.8 ± 0.2; *p* = 0.49) and hADSCs (0.7 ± 0.1; *p* = 0.58) groups compared with the sphincterotomy group. The immunohistochemical findings followed the same pattern (Fig. 3).

### MHC gene expression

**Fig. 4:**
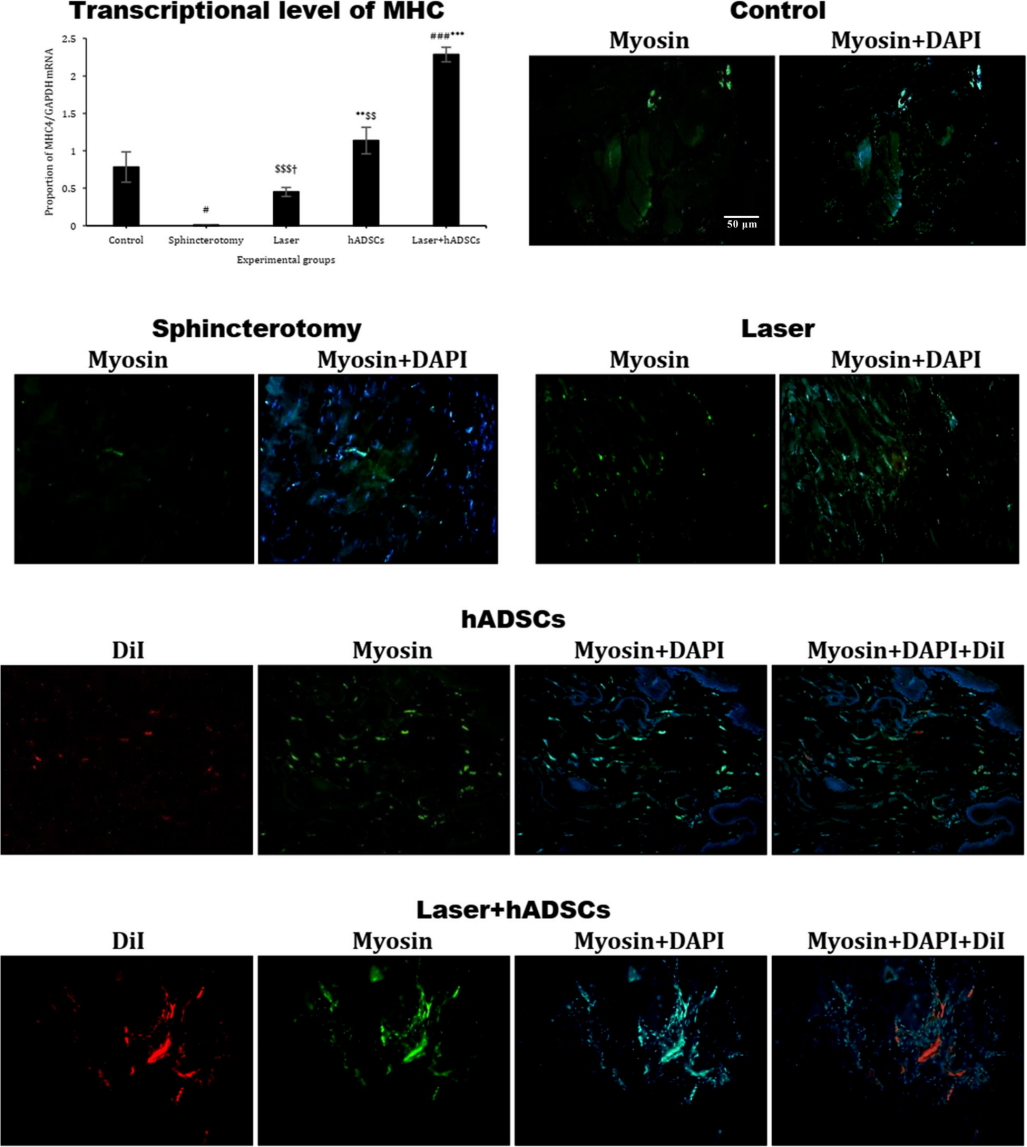
Gene and protein expression of myosin in injury site of the external anal sphincter after laser, human adipose-derived stem cells (hADSCs), and combination of laser and hADSCs (laser + hADSCs) treatments. hADSCs labeled by DiI (red). Myosin expression is observed in the hADSCs and laser groups (green). Data are presented as means ± SEM (*n* = 4 animal per group). ***Significant level at *p* < 0.0001 with the sphincterotomy group. **Significant level at *p* < 0.01 with the sphincterotomy group. ^###^Significant level at *p* < 0.0001 with the control group (intact animals). ^$$$^Significant level at *p* < 0.0001 with the laser + hADSCs group. ^$$^Significant level at *p* < 0.01 with the laser + hADSCs group. ^†^Significant level at *p* < 0.05 with the hADSCs group. Real-time PCR, immunohistochemistry, and western blotting, external anal sphincter, rabbit (× 10)

Like actin gene expression, MHC mRNA gene expression was reduced with sphincterotomy (df = 4, *F* = 43.5; *p* < 0.0001). Both the hADSCs (1.1 ± 0.2; *p* = 0.001) and laser + hADSCs (2.3 ± 0.1; *p* < 0.0001) groups had increased MHC expression compared with the sphincterotomy group (0.01 ± 0.003), and the laser + hADSCs group had greater MHC mRNA gene expression than either the laser (0.4 ± 0.05) or hADSCs group (*p* < 0.0001). Finally, the hADSCs group had greater MHC gene expression than the laser group (*p* = 0.04). Immunohistochemical findings followed the same pattern (Fig. 4).

### VEGFA gene expression

**Fig. 5:**
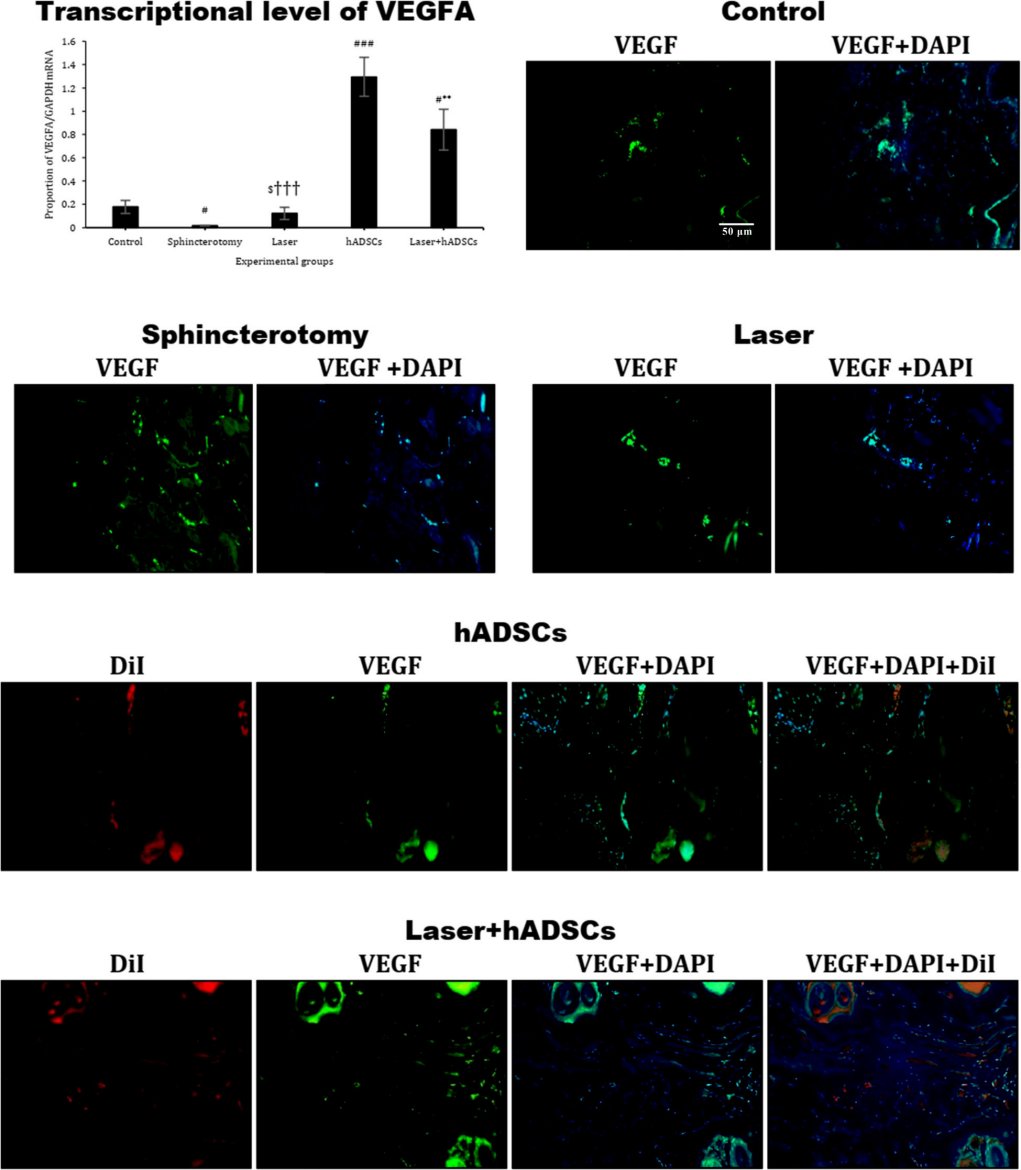
Gene and protein expression of VEGF in injury site of the external anal sphincter after laser, human adipose-derived stem cells (hADSCs), and combination of laser and hADSCs (laser + hADSCs) treatments. hADSCs labeled by DiI (red). VEGF expression is observed in the hADSCs and laser groups (green). Data are presented as means ± SEM (*n* = 4 animal per group). **Significant level at *p* < 0.01 with the sphincterotomy group. ^###^Significant level at *p* < 0.0001 with the control group (intact animals). ^#^Significant level at *p* < 0.05 with the control group. ^$^Significant level at *p* < 0.05 with the laser + hADSCs group. ^†††^Significant level at *p* < 0.001 with the hADSCs group. Real-time PCR, immunohistochemistry, and western blotting, external anal sphincter, rabbit (× 10)

VEGFA gene expression was reduced 3 months after sphincterotomy (df = 4, *F* = 23.7; *p* < 0.0001). The hADSCs (1.3 ± 0.2; *p* < 0.0001) and laser + hADSCs (0.8 ± 0.2; *p* = 0.005) groups had increased VEGFA mRNA gene expression compared with the sphincterotomy group (0.02 ± 0.003). The laser + hADSCs group had greater VEGFA expression than the laser group (0.1 ± 0.05; *p* = 0.01) but did not differ from the hADSCs group (*p* = 0.18). The hADSCs group also had greater VEGFA expression than the laser group (*p* < 0.0001). Immunohistochemical findings again followed the same pattern (Fig. 5).

### Vimentin gene expression

**Fig. 6:**
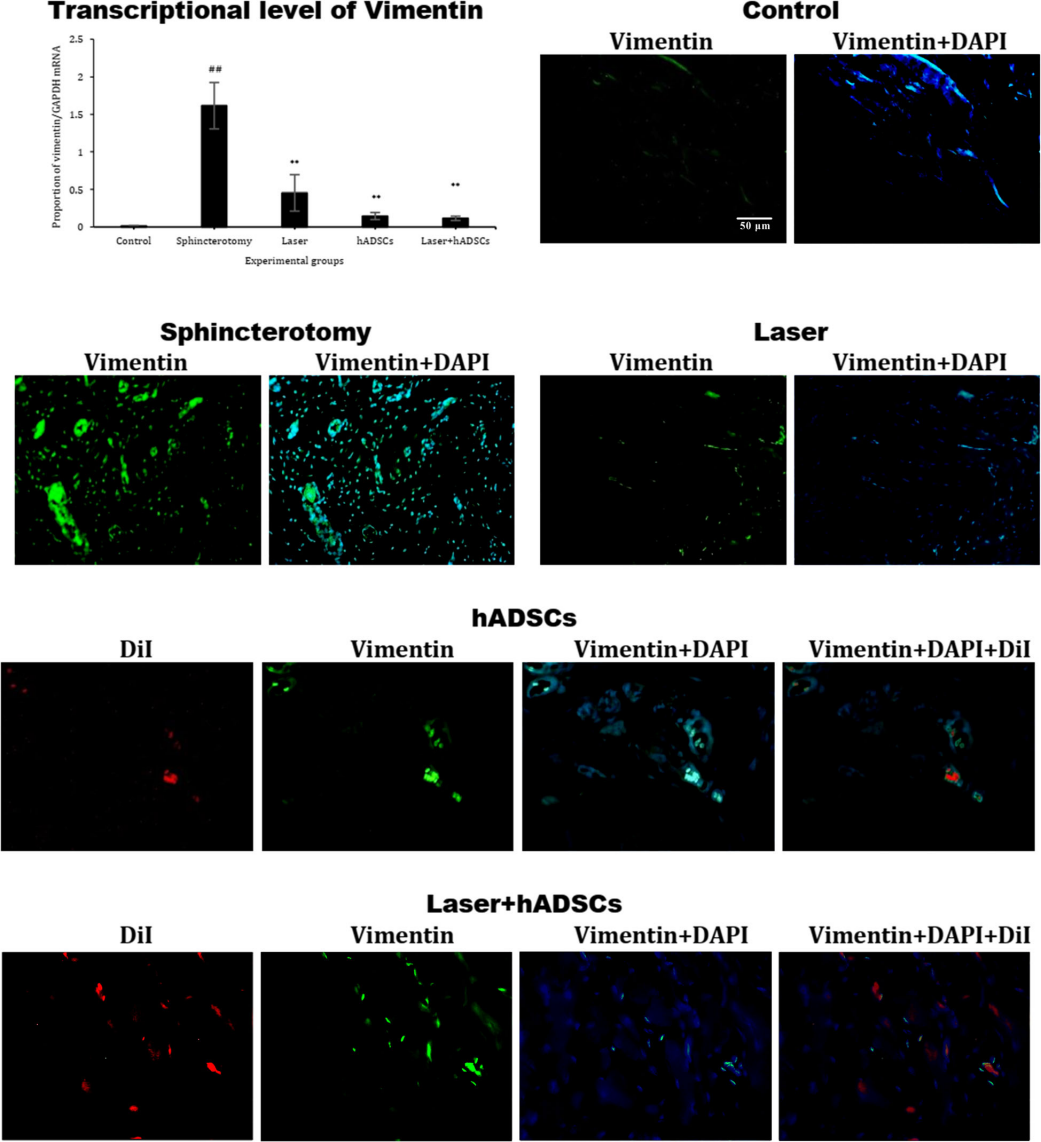
Gene and protein expression of vimentin in injury site of the external anal sphincter after laser, human adipose-derived stem cells (hADSCs), and combination of laser and hADSCs (laser + hADSCs) treatments. hADSCs labeled by DiI (red). Vimentin expression is observed in the hADSCs and laser groups (green). Data are presented as means ± SEM (*n* = 4 animal per group). **Significant level at *p* < 0.01 with the sphincterotomy group. ^##^Significant level at *p* < 0.01 with the control group (intact animals). Real-time PCR, immunohistochemistry, and western blotting, external anal sphincter, rabbit (× 10)

The vimentin mRNA gene expression was increased after sphincterotomy (df = 4, *F* = 13.9; *p* < 0.0001). The hADSCs (0.1 ± 0.05), laser (0.4 ± 0.2), and laser + hADSCs (0.1 ± 0.03) groups had decreased vimentin expression compared with the sphincterotomy group (1.6 ± 0.3; *p* < 0.0001); such expression was similar to the control group (0.01 ± 0.002; *p* > 0.99). Immunohistochemical findings followed the same pattern (Fig. 6).

### Ki67 gene expression

**Fig. 7:**
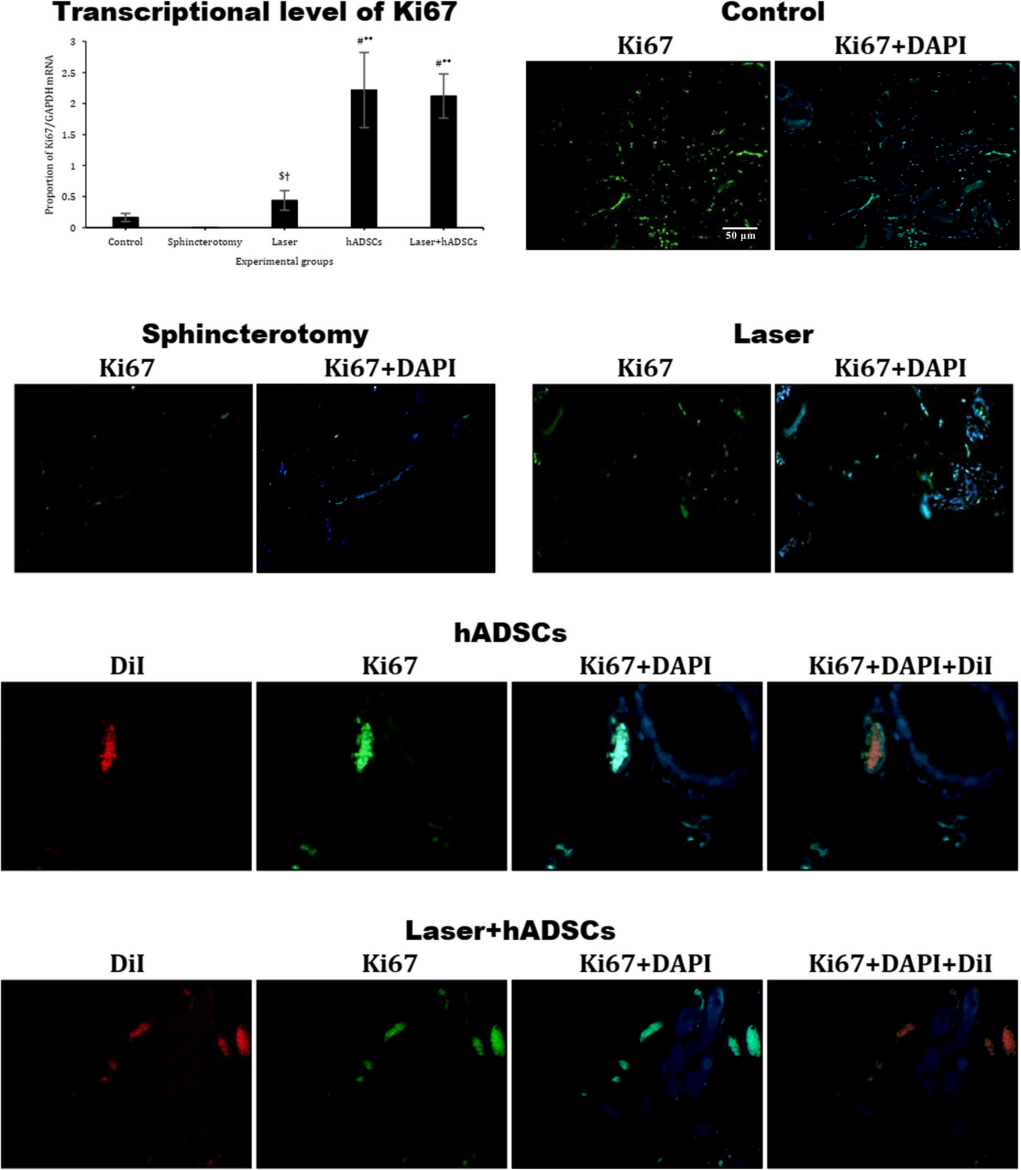
Gene and protein expression of Ki67 in injury site of the external anal sphincter after laser, human adipose-derived stem cells (hADSCs), and combination of laser and hADSCs (laser + hADSCs) treatments. hADSCs labeled by DiI (red). Ki67 expression is observed in the hADSCs and laser groups (green). Data are presented as means ± SEM (*n* = 4 animal per group). **Significant level at *p* < 0.01 with the sphincterotomy group. ^#^Significant level at *p* < 0.05 with the control group (intact animals). ^$^Significant level at *p* < 0.05 with the laser + hADSCs group. ^†^Significant level at *p* < 0.05 with the hADSCs group. Real-time PCR, immunohistochemistry, and western blotting, external anal sphincter, rabbit (× 10)

Although sphincterotomy (0.005 ± 0.001) had no effect on Ki67 mRNA gene expression (*p* > 0.99), the hADSCs (2.2 ± 0.6; *p* = 0.007) and laser + hADSCs (2.1 ± 0.4; *p* = 0.009) groups showed greatly increased Ki67 expression compared with controls (0.2 ± 0.1) (df = 4, *F* = 11.4; *p* = 0.001) (Fig. 7).

## Discussion

The findings of the present study showed that the combination of laser and hADSC therapy improved anal sphincter function after experimental injury more than either treatment alone. The efficacy of the combination therapy appears to relate to an increase in the number of motor units, decreased ratio of scar tissue to myofibril content, and an increase in the expression of myogenic, angiogenic, and proliferative markers.

The relative contribution of the EAS and IAS to resting anal pressure is typically about 15% and 85% respectively, while maximum squeeze pressure is mainly generated by the EAS [26]. In our study, both resting and maximum squeeze pressures were greatly decreased after sphincterotomy compared with the control group [25]. However, the resting pressure of the anal sphincter was improved with either laser or hADSC therapy, and it was more prominent in combination therapy. Laser treatment can induce muscle repair through satellite cell proliferation and differentiation [27], through increase in myofibril numbers [28], through anti-apoptotic [27] and anti-inflammatory [29] properties, and by stimulating angiogenesis [17]. Our results confirmed that laser therapy aids muscular repair via increased ki67 (proliferation factor) and VEGF mRNA gene expression compared with the sphincterotomy group. Mitochondria are recognized as the main photoreceptors within the cell, and the abundance of mitochondria in skeletal muscle (EAS) compared to smooth muscle (IAS) explains the different effects of laser therapy on the two sphincters [30]. Ehrreich et al. reported in 1968 that smooth muscle (other than vascular smooth muscle) exhibits very little sensitivity to visible and ultraviolet radiation [31]. This is consistent with our finding that resting pressure increase in the laser group is less than that in the hADSCs group. By contrast, hADSCs have the potential to repair both skeletal and smooth muscles, so the hADSCs group displayed higher resting pressure hADSCs compared with the laser group.

The fact that laser therapy promotes migration, proliferation, paracrine activity, and differentiation of hADSCs [32–34] explains why the combination therapy had a greater increase in resting anal pressure than hADSCs alone. Our immunohistochemistry and mRNA expression findings showed more expression of α-actin and myosin heavy chain, in the combination group than the laser or hADSCs alone. Escalating effects of the laser on Ki67 and VEGFA expression could be due to their short half-lives (1 to 1.5 h [35] and 30 to 45 min [36] respectively). As a result, a difference in expression of these indicators of proliferative and paracrine activity might have been observed, if hADSC gene expression had been examined immediately after laser therapy at the end of the second week.

We found that the laser, hADSCs, and combination groups had increased maximum squeeze pressure compared with the sphincterotomy group, without any differences between them. The maximum squeeze pressure is the result of the EAS, despite of the resting pressure, which is the result of both EAS and IAS. There is no significant difference among the effects of the laser, hADSCs, and laser + hADSCs on EAS. If the maximum squeeze pressure (similar to the resting pressure) came from the contractility tone of the two muscles, the same results would be expected.

Our EMG findings showed that sphincterotomy dramatically decreased the number of motor unit numbers compared with the control animals, consistent with our observations of a high collagen content at the site of injury. The laser, hADSCs, and combination therapy groups showed a significant increase in motor units, consistent with a surge of myofibrils in the injured areas and a reduction in collagen content. Both Roth et al. and Weiss et al. demonstrated that injured areas of muscle were filled with new myofibrils capable of contractile activity [28, 37]; additionally, Shefer et al. showed that laser irradiation could stimulate the satellite cells near myofibrils and form new myofibrils for muscular repair [27]. Moreover, laser irradiation could modulate the collagen content in injured areas through nuclear factor kappa-B (NF-kB) gene expression, collagen I and III remodeling [38], and a reduction in collagen content in skeletal muscles [39]. Rodriguez et al. indicated that the hADSCs could repair a posterior tibialis muscle injury via formation and fusion of new myotubes [40]. Liu et al. ascertained that hADSCs express the myogenic biomarkers (MyoD, myogenin, and MYH) which could differentiate hADSCs to myotubes. They also showed the myotube fusions and hADSC contribution in muscular repair [41]. Pecan et al. demonstrated that hADSCs could increase myofibril numbers in injured areas. This results from both differentiation of hADSCs into myogenic lines and the activation, division, and differentiation of satellite cells through hADSC paracrine activity, including IGF-1, VEGF, and HGF secretion [42]. In addition to the myofibril formation at the site of the lesion, other paracrine activities of hADSCs (regulation of TGF-β1 and TGF-β2 via HGF secretion) have anti-fibrotic effects, reducing collagen deposition at the site of the muscle lesion [23, 43].

In our study, motor unit numbers in the combination group were higher than those in the laser treatment alone. This may be due to the additive effect of the laser and hADSCs on collagen content reduction and myofibril surge. In the hADSCs treated group, the motor unit number increased. It may be related to the role of stem cells in muscle healing, either differentiating into muscle cells, having anti-fibrotic effects, or promoting and facilitating satellite cell differentiation by other mechanisms [27–29]. The results of immunohistochemistry and gene expression markers for skeletal muscles (MHC and α-actin) have confirmed these effects.

Along with the collagen synthesis, a key step in wound healing is the remodeling of collagen, in which the biomechanical properties of the scar are optimized [44]. Remodeling is usually initiated 2 to 3 weeks after injury and continues for more than 1 year [45]. Vimentin is an intermediate filament that is necessary for the collagen formation and remodeling in response to injury [44, 46]. In the present study, the highest collagen and vimentin expression was seen in the sphincterotomy group, which may relate to vimentin’s role in the remodeling of collagen. However, vimentin expression during the regeneration stage of the skeletal muscle is inversely proportional to the number of mature myofibrils. It means that intact skeletal muscle with myotube cross-linking and mature myofiber lacks vimentin expression [47]. In the present study, the strong reduction in vimentin expression in the laser, hADSCs, and combination groups may indicate mature myofibrillar replacement in the injured areas. The resting and maximum squeeze pressures, expression of specific skeletal muscle genes (α-actin and MHC), EMG, and collagen content assessment are consistent with a significant surge of mature myofibrils.

In our study, hADSCs were used. Some animal studies show that xenograft transplantation of mesenchymal stem cells can reduce the immunomodulation [48] and efficacy of these cells [49]. However, the use of hADSCs has been usual in preclinical studies [50–52] and its efficacy is proven. The findings of the present study also show that xenograft transplantation of hADSCs in the anal sphincterotomy model in rabbit improves sphincter function and tissue repair. It is recommended to compare the efficacy of xenograft and allograft transplantation of ADSCs in future studies.

A fourth-degree anal sphincter injury usually occurs after a vaginal delivery, iatrogenic injury, pelvic injury in a motor vehicle accident, or blast injury. hADSC preparation and transplantation is a time-consuming process. Therefore, the hADSCs could not be an ideal option for acute-phase injury. However, laser therapy is a non-invasive and more suitable method in acute anal sphincter injuries.

The range of light used in LLL ranges from 600 to 1070 nm. The effect of different wavelengths of a laser varies depending on the site and depth of injury. In our sphincterotomy model, the skin, mucosa, and muscle were incised. According to the study of Chung et al. [53], the optimum laser wavelength in repairing superficial tissues is 600 to 700 nm. Therefore, like many other articles on muscle repair [54–56], we used a 660-nm laser. The reason for the selection of the 660-nm wavelength is optimum efficacy in inflammatory processes, angiogenesis, fibroblast proliferation, and cytokine secretion, while such effects are not observed at higher wavelengths (940 and 808 nm) [57].

Although we confirmed the survival of transplanted cells at the end of 90th day, the survival rate was not compared in the different groups and the effect of LLL on hADSC survival in the combination group was not clear. Laser is a non-invasive treatment compared to cell therapy. However, the effect of laser on some parameters such as anal sphincter resting pressure is less than that of hADSCs. We suggest that different protocols for laser irradiation including different durations of the laser irradiation in each session or shorter/longer wavelengths be studied in future trials. We measured the squeeze pressure when the probe was inserted into the animal rectum, and then we stimulated the external anal sphincter using the tingling method. Although recording squeezing pressure during EMG stimulation is a better method, we have used the tingling method to mimic a natural stimulus (such as coughing or striking the external anal sphincter) that causes squeeze pressure of the anal sphincter. Therefore, lack of a significant difference between the treated groups may be due to the method of squeeze pressure measurement. Overall, it seems that the measurement of squeeze pressure in animal studies is not reliable.

## Conclusion

The present study indicates that co-application of laser and hADSC therapy immediately after anal sphincter damage could improve subsequent sphincter function. Moreover, combined therapy appears more effective for regaining resting anal pressure, and stimulating myogenesis and angiogenesis, than either laser or hADSCs alone.

## Supplementary information

**Additional file 1: Table S1.** Primer sequences for real time PCR. **Figure S1.** Flow cytometry of hADSCs. Morphology (fifth passage) (A) and cell surface markers (B-D) use for characterization of hADSCs (CD29+, CD73+, CD105+, CD34-, and CD45-).

## Data Availability

The data that support the findings of this study are available from the corresponding author upon reasonable request.

## Abbreviations

ACTA1: Skeletal muscle alpha-actin
EAS: External anal sphincter
EMG: Electromyography
GAPDH: Glyceraldehyde-3-phosphate dehydrogenase
hADSCs: Human adipose-derived stem cells
LLL: Low-level laser
IAS: Internal anal sphincter
MUAP: Motor unit action potential
MYH: Myosin heavy chain
VEGF: Vascular endothelial growth factor A
