# Supplementary material for: Combination of laser and human adipose-derived stem cells in repair of rabbit anal sphincter injury: a new therapeutic approach
Source: Stem Cell Res Ther. 2019 Dec 2;10:367. doi: 10.1186/s13287-019-1477-5 (PMC6889595; doi:10.1186/s13287-019-1477-5)
Supplement: Supplementary file 1 — Additional file 1: Table S1. Primer sequences for real time PCR. Figure S1. Flow cytometry of hADSCs. Morphology (fifth passage) (A) and cell surface markers (B-D) use for characterization of hADSCs (CD29+, CD73+, CD105+, CD34-, and CD45-). [file 13287_2019_1477_MOESM1_ESM.pdf]

Supplement table 1: Primer sequences for real time PCR

Ptrimer name	Sequence 5´→3´
ACTA1	
Forward	GATGAAGGAGGGCTGGAAGA
Reverse	GACTTCGAGAACGAGATGG
MYH	
Forward	CTGGCTGGCTGGACAAGAA
Reverse	TGAAGAGAGCAGACACGGTC
Vimentin	
Forward	GACAGGATGTTGACAATGC
Reverse	GTCGATCTGGACATGCTGTT
VEGFA	
Forward	TGCTGTAGGAAGCTCATCTC
Reverse	GGCTGCTGCAATGATGAAAG
Ki67	
Forward	AAGGACTGGAAATAGCAGAGG
Reverse	GGATGTGATGGCTGATGAA
GAPDH	
Forward	CTAGAGCAACAGGGTGGTGG
Reverse	AGCGTGGTGGGACTGAGTGG

ACTA1: Actin, alpha 1, skeletal muscle; GAPDH: Glyceraldehyde-3-phosphate dehydrogenase; MYH:Myosin heavy chain ; VEGFA: Vascular endothelial growth factor A

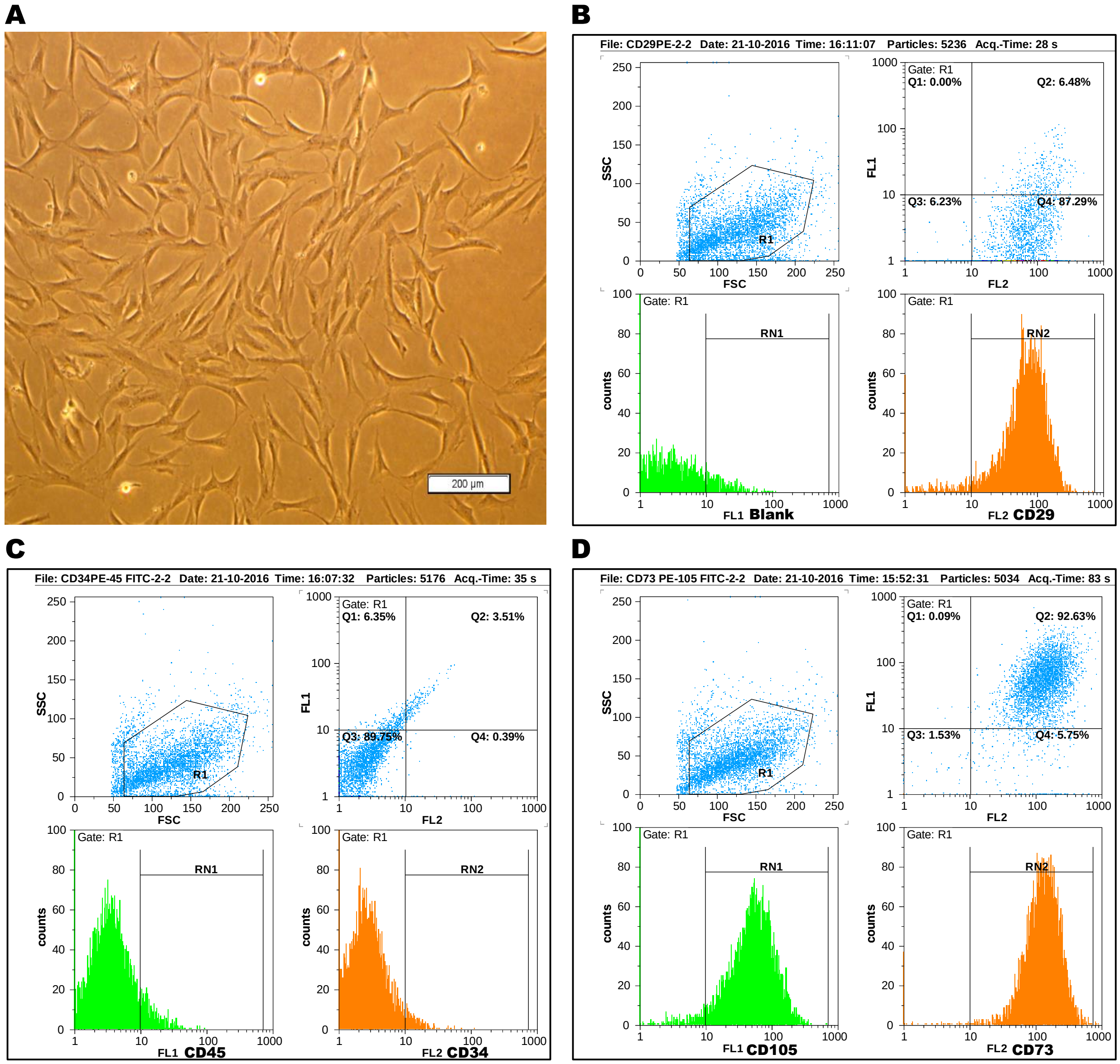

Supplement figure 1: Flow cytometry of hADSCs. Morphology (fifth passage) (A) and cell surface markers (B-D) use for characterization of hADSCs (CD29$^+$, CD73$^+$, CD105$^+$, CD34$^-$, and CD45$^-$).
